# AGE-RELATED ELEVATED CD4+ T HELPER 17 CELL RESPONSE PROMOTES PROSTATE CANCER CELL GROWTH, MIGRATION, AND INVASION

**DOI:** 10.1093/geroni/igz038.3221

**Published:** 2019-11-08

**Authors:** Qiuyang Zhang, Sen Liu, Bing Zhang, Elizabeth Norton, S Michal Jazwinski, Oliver Sartor, Chad Steele, Asim B Abdel-Mageed

**Affiliations:** 1 Tulane University School of Medicine, New Orleans, Louisiana, United States; 2 Tulane University School of Medicine, New Orleans, United States

## Abstract

Age is the most important risk factor for prostate cancer (PCa). But, how age contributes to PCa remains unknown. Interleukin-17 (IL-17) -producing CD4+ T helper 17 (Th17) cells play a critical role in inflammatory diseases. It is often elevated in aging humans and mice, however, whether aging affects Th17 cell function and subsequent PCa risk increase is unclear. In this study, we investigated the role of CD4+ T cells in PCa cell growth during the aging process. Splenic T cells were isolated and purified into CD4+CD25- T cells from young and old mice, then cultured in the presence of plate-bound anti-CD3/anti-CD28. Four days later, the cells were re-stimulated with PMA and ionomycin in the presence of brefeldin A for 4 hours and then were collected and used for flow cytometry and/or qPCR. The supernatant (conditioned media) from young and old cultures was collected and used in subsequent experiments. Flow and qPCR results showed that 17-producing T cells and associated cytokines were significantly increased in old mice compared to young mice. When PCa cell lines (LNCaP, DU-145, and PC3) were treated by the conditioned media for 48 and 72 hours. The cell proliferation, migration, and invasion, as well as the activation of NF-B signaling in PCa cells, were significantly increased after exposure to the conditioned media from aged mice, compared to that from young mice. These results indicated that age-related CD4+ Th17 cell responses are elevated in mice in the aging process and play an important role in PCa growth.

